# Conditional Random Field-Based Offline Map Matching for Indoor Environments

**DOI:** 10.3390/s16081302

**Published:** 2016-08-16

**Authors:** Safaa Bataineh, Alfonso Bahillo, Luis Enrique Díez, Enrique Onieva, Ikram Bataineh

**Affiliations:** 1 Faculty of Engineering, University of Deusto, Av. Universidades, 24, Bilbao 48007, Spain; alfonso.bahillo@deusto.es (A.B.); luis.enrique.diez@deusto.es (L.E.D.); enrique.onieva@deusto.es (E.O.); 2 DeustoTech-Fundación Deusto, Fundación Deusto, Av. Universidades, 24, Bilbao 48007, Spain; 3 Department of Architecture, Jordan University of Science and Technology, Irbid 22110, Jordan; ikrambataineh@yahoo.com

**Keywords:** offline map matching, indoor positioning, indoor localization, navigation systems, conditional random fields, map modelling, inertial sensors, mobility of pedestrians

## Abstract

In this paper, we present an offline map matching technique designed for indoor localization systems based on conditional random fields (CRF). The proposed algorithm can refine the results of existing indoor localization systems and match them with the map, using loose coupling between the existing localization system and the proposed map matching technique. The purpose of this research is to investigate the efficiency of using the CRF technique in offline map matching problems for different scenarios and parameters. The algorithm was applied to several real and simulated trajectories of different lengths. The results were then refined and matched with the map using the CRF algorithm.

## 1. Introduction

The importance of indoor navigation arises from the necessity of context-aware services that work inside buildings, given that people spend most of their time indoors [1]. Although outdoor navigation appeared prior to indoor navigation, the former techniques cannot be applied directly to the indoor environment. This is firstly associated with the fact that outdoor navigation systems mostly depend on Global Navigation Satellite Systems (GNSS) that function poorly indoors due to multipath and attenuation losses, and secondly because pedestrian indoor movement is limited by building structure and details that are totally different from those found on road maps [2].

As indoor environments possess spatial constraints that can be used to rule out certain incorrect localization results, indoor maps have become an additional data source with which to improve the accuracy and reliability of indoor localization systems. For instance, it is unreasonable that an estimated walking trajectory passes through an area occupied by an obstacle such as a wall. The process of utilizing map information in the localization processes of objects or persons is known as map matching. Map matching can be used to find the correspondence between a sequence of points representing the walking trajectory obtained via a localization system and a given map.

In outdoor transportation systems, map matching techniques vary from simple geometric search methods to advanced complex probabilistic models [3]. Similarly, existing indoor map matching techniques can be geometric, topological, or probabilistic. The differences between indoor and outdoor map matching problems lie in the complex structure of indoor maps compared to road maps, as well as in the randomness of pedestrian behavior compared to that of vehicles [2].

Indoor map matching techniques that use advanced probabilistic models are usually based on recursive Bayesian models such as Hidden Marcov Models (HMMs) and Particle Filters (PFs) [4,5,6,7]. However, recursive Bayesian models involve computing the joint probability distribution between all states and observation variables, which makes them computationally expensive. The Conditional Random Fields (CRFs), a probabilistic model that is widely employed in speech recognition, can also be used in map matching [8,9]. It is a sequence-labelling algorithm that employs the observations and context information as features to evaluate the conditional probability of state transitions. CRF is based on calculating conditional probability which makes it computationally more efficient than Bayesian models, besides being more flexible in handling arbitrary dependencies between observations [10]. However, CRF requires a backward phase in order to evaluate different path alternatives, which delays the process and makes it less suitable for real-time applications. On the other hand, offline (Offline localization systems: In localization systems, the expression “offline” has been used in the literature with two different meanings: (1) those localization systems that are not real time, also known as global systems, in which measurements are taken for the whole walk and an offline algorithm is then used to estimate the track; and (2) those localization systems that use local resources and sensors without having been connected to any global system or central server. In this paper, we use the term to refer to the first meaning.) (i.e., not real time) map matching can allow for reduced performance in favor of accuracy [11]. Offline map matching results can be used for both post-processing analysis and modelling purposes. Many indoor applications that need behavioral analysis and data mining can make use of offline map matched trajectories.

CRF was applied for the first time in pedestrian indoor localization by Xiao et al. in their localization system MapCraft [12]. In MapCraft, the CRF map matching algorithm is tightly coupled to the localization system that takes raw measurements directly from sensors such as Wi-Fi, Bluetooth and inertial sensors as inputs to the algorithm, and fuses it together with the map information. What we mean by what we call ‘tight coupling’ is that the CRF algorithm here is used to fuse all measurements and spatial map information altogether at the same time; both positioning and map matching are done together in one module. MapCraft aims to be used as real time map matching system; however the delay produced by the backward phase of CRF is a real challenge that affects the efficiency of the algorithm when used for real-time tracking; In MapCraft they overcome this problem by making the compromise of converting the conditional probability discrete distribution obtained by the forward phase to a Gaussian distribution and displaying it on the map in real-time [12]. This means less accuracy as it does not give the optimal solution which can only be obtained in the backward phase; that means that the CRF was not implemented completely in MapCraft for the online mode. We do not know exactly to what degree that step affected the accuracy of their system when used online. Nevertheless, their step can be a good example of that CRF is more suitable for offline usage. As they mentioned clearly in their paper, the optimal path can only be calculated “in the case of delay tolerant offline tracking” [12].

Regarding offline tracking and trajectory detection, it is possible to apply the CRF can be directly to the raw sensors’ measurements to estimate the location and match it with map; however this makes the map matching dependent on the environment and highly customized, as the types of the available sensors should be known; or we can separate the map matching part from localization; we can take location trajectories obtained by any localization system and then match it with the map offline; the map matching system will be independent of the sensors types and of the localization system, and can be attached to (coupled with) any existing localization system as an independent module, separate from the localization module; this modular structures means more flexibility.

In this paper, we present an offline CRF map matching algorithm that can be easily loosely coupled to any existing localization system; it uses only the output estimated walking trajectory of the localization system as an input and refines it based on only the map information, without the direct use of raw sensor data. The adoption of loose coupling means that the map matching algorithm does not need to know the implementation details of the primary localization system; this also then allows the map matching algorithm to be linked to any localization system as a separate module and to use only its output. Any change in the localization system will not affect the implementation of the map matching algorithm.

An example of using CRF as an offline map matching algorithm for outdoor systems can be found in the work of Xu et al. [13]; in their work they use the GPS timestamped locations generated by the vehicle as input trajectory for the map matching algorithm and the output is a sequence of road segments traversed by the vehicle. As an outdoor map matching algorithm, the map model is based on road segments. In our work with indoor systems, the map structure is different from the road network, we used a grid based map model that divide the 2D map to squared cells. Our transition model is based on the neighborhood between cells and the distribution of obstacles in the building, where in outdoor environments the transition is dependent on the road network and topology. Instead of using GPS locations as input, we use locations produced by a local positioning system as input as the GPS is not effective inside buildings.

We will explain in details in Section 2.2 how the CRF algorithm functions; but for the purpose of comparison we can say in brief that the CRF employs observations and context information through features functions that relates the observation with state transitions. In MapCraft, they used three feature functions depending on observations from sensors: (1) the first function uses displacement and heading measurements from the inertial sensors; (2) the second function is to handle correlations in heading errors of the inertial sensors; and (3) the third function uses the signal strength observation in conjunction with fingerprint map [12]. In our map matching CRF algorithm, we do not use observations from sensors as this supposed to be done by a pre-existing localization system; we only make use of the spatial context provided by the map as presented by the model in addition to the coordinates generated by the localization systems as observations. We use one feature function that makes use of the map information and the input coordinates.

The rest of paper is organized as follows: Section 2 describes the system architecture and components, as well as the proposed CRF model; the experimental results are presented and discussed in Section 3; and finally the conclusions drawn from this research are presented in Section 4.

## 2. Overview of the System

**Figure 1:**
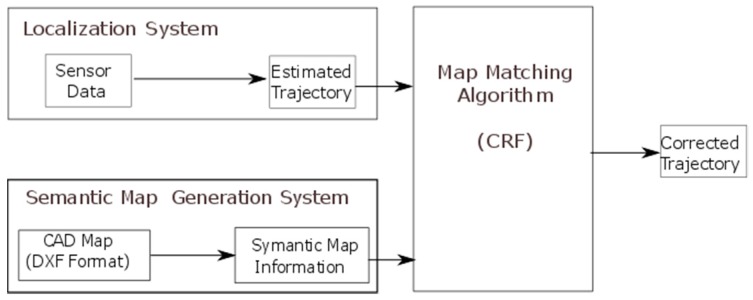
System Architecture, illustrating the coupling between the proposed map matching algorithm and the localization and semantic map systems.

In this section, the global architecture of the system is presented, as well as the implemented algorithm. First, we give a brief description of the system architecture, components and behavior; further details follow in Section 2.1 and Section 2.2. The system architecture is illustrated in Figure 1. As can be seen from this figure, the system is composed of three subsystems, with some interconnections between them. The three components of the system are:

1. The primary localization system that produces the first estimated trajectory. This can be any localization system that produces, as its output, a walking trajectory in the form of a time sequence of estimated location coordinates. This trajectory will be the input of the map matching algorithm.
2. The semantic map generation system, a unit that models the floor plan obtained from CAD files in a semantic format that can be used by the map matching algorithm. In our case, a grid-based map model was employed in which the floor plan map is divided into square uniform-grid cells. Each cell is associated with a semantic representation of its contents, for example if it contains a wall or a free space.
3. The map matching algorithm that refines the estimated trajectory (path) using the CRF technique and the semantic floor plan information.

System operation proceeds as follows: once the primary estimated trajectory is obtained by the localization system, map matching is carried out by applying the CRF model that also uses the information obtained from the map to produce a refined (corrected) trajectory. The goal is to estimate the most feasible trajectory, taking into account the constraints provided by the map such as walls and other obstacles that are obtained from a semantic map generation system, a separate unit that extracts map data from CAD files and represents it in a model so it can be used by the algorithm.

### 2.1. The Semantic Map Generation System

Indoor maps can be available in different formats, like images, PDF files, or CAD files. These formats are not suitable to be directly used by the map matching algorithm; two tasks should be performed, the map information should be extracted from the available map files, then this information should be represented in another format usable by the map matching algorithm. The most commonly used map models in indoor localization are the grid models and the graph models [2]. In the grid models, the space is partitioned into regular cells with semantics. The graph models [14] reconstruct the space as a graph where nodes represent entities or places of interest in the building. In [12], for example, they obtained graphs from maps in image formats using standard edge detecting algorithms to extract edges from the image, then a grid-based map model was used with cell size of 0.8 m to model the map information. In our system, we preferred to use the CAD files because CAD is very widely used in architectural design, and we modelled the map using a grid-based model.

The semantic map generation unit extracts the map data from the CAD files and models it in a semantic model suitable for the map matching algorithm. We use the CAD data encoded in the Drawing Interchange File format (DXF). DXF files are standard ASCII text files that are offered by CAD and that can be easily read by other programs [15,16]. However, map information obtained from DXF files is not suitable for localization applications, as it is not enhanced with semantic information that allows computers to understand the architectural structure of the building and to distinguish between different architectural objects such as walls and stairs [17]. Instead, DXF-derived map information comprises only line, curve, circle, and polyline drawing data, which means that it requires more processing in order to be used by the proposed map-matching algorithm. We extract the DXF information and represent it using our map model, which is based on dividing the map into square cells and determining the possible transitions of the pedestrian from one cell to another depending on the existing obstacles. We extract the simple entity information from the DXF files and locate it in the grid cell representation model of the floor plan.

**Figure 2:**
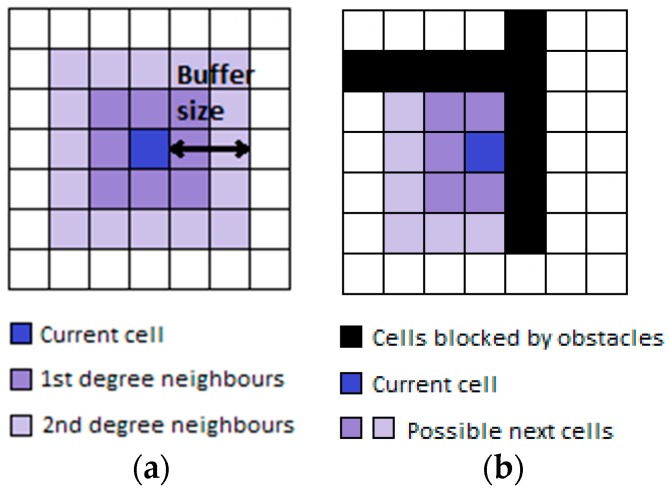
Search region for the next location, including allowed transitions in the next step (**a**) and allowed steps in the presence of obstacles (**b**).

As we will explain in Section 2.2, the CRF model is a classifier that requires pre-defined states/labels. To this end, the proposed map model is designed to suit this specific purpose by representing the map as a group of square cells, with each cell representing a squared area in the building; to the algorithm, this represents a state/label that can be used to specify the position of the pedestrian. In the model, each cell has its own characteristics, be it representing a free space in the building or be it occupied by an obstacle such as a wall or furniture. For the purpose of representing state transitions, each cell knows its neighbor cells and the neighbors of neighbor cells. A transition graph/table is generated in such a way that the transition is only possible between neighbor cells that do not contain any obstacle; for more flexibility, a transition to a neighbor of a neighbor cell is also allowed under the condition that there are no obstacles impeding that transition. The search region for the next location from the current location is known as the buffer [2], with the maximum transition distance allowed in each direction known as the buffer size. Allowing transitions to neighbor cells and neighbors of neighbors results in a buffer size of 2 cells in each direction, as shown in Figure 2a. Figure 2b presents an example of possible transitions based on the existence of obstacles.

### 2.2. The Map Matching Algorithm

**Figure 3:**
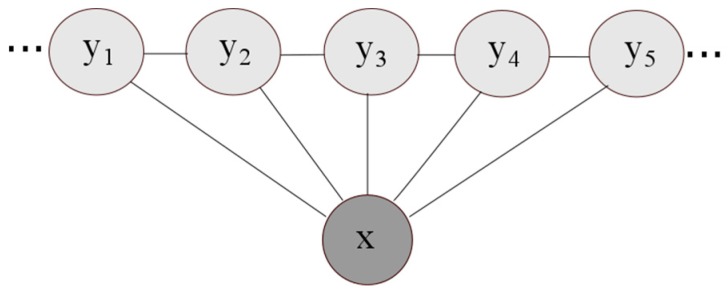
Linear Chain CRF model (adapted from [8]).

A CRF model is used as the base model in the developed system. As undirected probabilistic graphical models developed for labelling data [8], CRF models are used for an input set of observations $\vec{x}$ to predict a vector of hidden variables $\vec{y}$. Unlike generative models such as HMMs that model the joint probability $P\left(\vec{x},\vec{y}\right)$ by applying Bayes’ rule, CRFs are discriminative models that model the conditional distribution $P\left(\vec{x}|\vec{y}\right)$ over the hidden variables $\vec{y}$ given observation vector $\vec{x}$; a comparison of generative and discriminative models can be found in [18]. In linear chain CRFs, a special form of CRF graphs that model the output variable as a sequence [9], the conditional probability of states given observations $P\left(\vec{x}|\vec{y}\right)$ is proportional to the product of potential functions that link observations to consecutive states. Figure 3 shows a representation of the linear chain CRF model. The hidden states $\vec{y}$ are dependent on input observation vector $\vec{x}$; each hidden state depends not on one input value, but rather on the whole input vector. As a result, they can be affected by input observations from different time steps; which is considered an advantage of the CRF technique.

In the map matching problem, the hidden state vector $\vec{y}$ represents the sequence of locations to be calculated, i.e., the corrected walking trajectory; our Linear Chain CRF algorithm uses the cells in the map model as the hidden states/labels. Vector $\vec{x}$ represents the system input that can be either coordinates of the locations on an estimated trajectory obtained by some localization system, as in the case of our algorithm, or direct measurements from sensors.

The CRF algorithm consists of two phases: the forward phase and the backward phase. Following the calculation of the conditional probabilities of all cells in all time steps during the forward phase, inference is carried out in the second phase (backward phase),with the optimal trajectory chosen from among different candidate trajectories.

During the forward phase, the CRF algorithm evaluates the possible transitions at each time step according to the input trajectory and the transition graph obtained from the map model; this involves calculating the probabilities of transition from all cells of the current time step to all cells of the next time step. A probability value is assigned to each cell in the map at each time step; this value represents how probable it is that the pedestrian is located in that cell at that time step (for example, the probability of pedestrian movement to a cell occupied by a wall is zero). This probability is also a conditional probability calculated using so-called feature functions that compute to what degree the input observations support the choice of a cell to be on the trajectory at that time step. However, cell selection depends not only on its probability value at the current time step, but also on the previous time steps, i.e., the path history, and how this cell is related to others in the path. Hence, choosing the cell with the highest probability is not enough; the probability of a whole trajectory should be calculated.

The feature functions specify how the transition between two states is supported by the set of observations $\vec{x}$. The potential function of a cell is calculated at each time step by calculating the exponential of the summation of all feature functions that support the selection of this cell multiplied by the transition probability of moving to this cell from the current cell at the current time step. The higher the potential function value, the higher the probability of the cell to be the next cell. At each time step, *j* the potential function is the exponential of the summation of all feature functions *f_i_* at that time step, and can be written as:

(1) $$\psi_{j}\left(\vec{x},\vec{y}\right)=\exp\left(\overset{m}{\underset{i=1}{\sum }}\lambda_{i}f_{i}\left(y_{j-1},y_{j},\vec{x}\right)\right)$$

where *m* is the number of features and $\lambda_{i}$ is the feature weight that can be determined by training the model. The conditional probability $P\left(\vec{x}|\vec{y}\right)$ of each cell is calculated by normalizing the potential function as follows:

(2) $$P\left(\vec{x}|\vec{y}\right)=\left(\frac{1}{Z\left(\vec{x}\right)}\right)\cdot \overset{n}{\underset{j=1}{\prod }}\psi_{j}\left(\vec{x},\vec{y}\right)=\left(\frac{1}{Z\left(\vec{x}\right)}\right)\cdot \exp\left(\overset{n}{\underset{j=1}{\sum }}\overset{m}{\underset{i=1}{\sum }}\lambda_{i}f_{i}\left(y_{j-1},y_{j},\vec{x}\right)\right)$$

where n is the number of output states/cells and Z is the normalization factor, with

(3) $$Z\left(\vec{x}\right)=\underset{\vec{x}}{\sum }\overset{n}{\underset{j=1}{\prod }}\psi_{j}\left(\vec{x},\vec{y}\right)\;$$

If N is the number of cells, then at every time step the potential functions should be calculated N × N times; however, knowing that transition can only happen between neighbor cells, one need only calculate the potential function of those neighbors. Thus, the calculation number at each step is O(N) and the complexity of the whole procedure is O(NT), where T is the number of time step observations.

For each observation, each cell stores only one conditional probability value (the highest), while the previous cell that gave this value is also stored as the best parent. This is necessary for the following inference step.

During the backward phase, inference is implemented in order to estimate the location over time, with the most likely sequence of hidden states calculated by maximising the sum of the conditional probability function. After the potential function for all cells is calculated for each observation, the optimal path is determined. This is carried out via a backward process using dynamic programming and the Viterbi algorithm [19]. The optimal path is obtained by maximizing the sum of conditional probabilities along the path:

(4) $$\vec{y}{\;}^{*}=\underset{\vec{y}}{\text{argmax}}\text{ P}\left(\vec{x}|\vec{y}\right).$$

Although this process might be assumed to be of high complexity, as we need to choose between all possible trajectories, it is actually linear because for each time step we only save one value of the conditional probability for each cell. Hence, the number of candidate paths equals the number of cells (N). The backward process is O (NT), as we start a backward process for each cell by determining the best parent, which is the neighbor cell with the highest conditional probability at the previous time step, before summing these conditional probabilities along the path until reaching the first parent in the path. The path with the highest sum is then chosen. The forward and backward phases of the map matching CRF algorithm are shown in Algorithm 1.

**Algorithm 1.** CRF Algorithm for the Map Matching Problem1**Input :** Observation = a vector of coordinates of the input estimated trajectory2**Output**: CorrectedPath = a vector of coordinates of the output corrected trajectory3**Forward Phase:**4For each observation (Observation_j_*) %Observation _j_ = coordinates of the input at time*
*step j*5**For** all cells (i)6    **For** all neighbor cells of i (k)7       f_dis_ = T(i,k)/Distance (k,Observation_j_)*%T(i,k):*
*transition possibility from cell i to cell k {0,1}*8       Potential(k,Observation_j_) = exp(f_dis_);9          Z = sum(Potential(:,j))  *%  normalization factor*10       ConditionalProbabilty (k,Observation_j_) = Potential(k,Observation_j_)/Z11    **If** (ConditionalProbabilty (i,Observation_j-1_) > ConditionalProbabilty (bestParent (k)) 12    **then** CorrectedParent(k) = i13    **End**14    **End**15**End**16**End**17**Backward Phase**18#CandidatePaths = #Cells19**For** p = 1➜ #CandidatePaths     *% p is the last cell in the CandidatePaths*20    k = p           *% k is the current cell in the CandidatePath*21    Construct each CandidatePath:22    **For** all observations (j ➜1)23       CandidatePath(j) = k; %add cell K to the path at time step j24       sum(CandidatePath) = sum(CandidatePath) + ConditionalProbabilty(k,j)25       k = BestParent(k,j) % choose the best parent of k to be the next cell in the path26**End**27**End**28CorrectedPath = CandidatePath with highest sum of ConditionalProbabilities

The main feature that we use in our CRF map matching algorithm is the Euclidean distance (in meters) between the centre of the candidate cell and the estimated position derived from the primary localization system; a smaller distance means a higher value of the potential function given that the transition to this cell is possible, as determined by the transition graph obtained from the map model. The proposed feature function can thus be defined as follows:

(5) $$f_{dis}=\frac{T\left(y_{j-1},y_{j}\right)}{Distance\;\left(x_{j},y_{j}\right)}\;\text{Minimum distance}=\text{cell size}/2$$

where T is a function that indicates the transition possibility from the current cell to the candidate cell, depending on the transition table, and which can be either 0 or 1, j is the time step and x is the current observation, which is the current location estimated by the primary localization system. This feature means that cells close to the current input locations have higher probabilities than far cells, on the condition that transition to the cell is possible from its neighbor cells and no obstacle forbids this transition.

## 3. Results and Discussion

The algorithm was tested using both simulations and data obtained from real measurements. Map information for the 4th floor of the DeustoTech building at the University of Deusto (Spain) was extracted from AutoCAD files and modelled as a grid-based map model. Section 3.1 presents the results of the conducted simulations, while the results obtained using the real measurements are presented in Section 3.2.

### 3.1. Simulations

**Figure 4:**
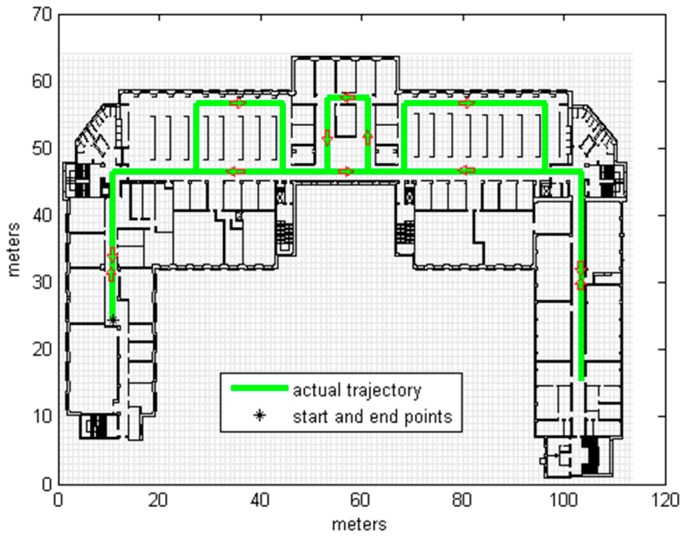
The Ground Truth, the arrows indicate the direction of walking.

A base trajectory (shown in Figure 4) was constructed as the ground truth, with different unmatched trajectories then created randomly as the input (observations) of the map matching algorithm. In real-life applications, the input trajectory is the output of an existing localization system that represents the primary estimations (as we will show in Section 3.2). The map matching system is applied to refine the estimated trajectory by avoiding crossing obstacles; the most feasible trajectory is the sequence of positions that violates the fewest constraints [12]. After matching the input trajectory to the map using the CRF algorithm, the matched (corrected) trajectory was compared to the ground truth in order to measure the precision, using the Cumulative Distribution Function (CDF) of the errors obtained by calculating the Euclidean distance between the actual position in the ground truth and the corresponding corrected position.

**Figure 5:**
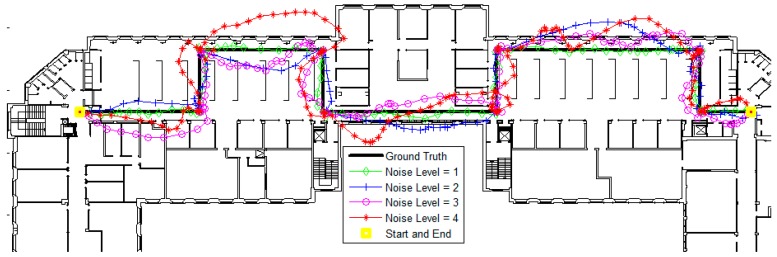
Trajectories with different noise levels.

To simulate different possible input trajectories with different qualities, different levels of noise were added to the base track by allowing different degrees of deviation (distances) and randomness around the ground truth. A low noise level was used to represent a primary estimation (input) trajectory with low error (mean error of about 1.3 m and standard deviation of about 1 m for noise level 1); a simulated input with a high noise level was used to represent an input trajectory with high error (mean error of about 2.5 m and standard deviation of about 1.8 m for noise level 4). Table 1 shows approximate values of the mean and standard deviations of the four different noise levels obtained by simulating 25 random samples of each noise levels. Examples of different simulated trajectories with different noise levels are shown in Figure 5.

The results of matching different input trajectories with the map are shown in Table 2. We can see that in all the 300 samples that we have run the number of obstacles crossed by the map matched trajectory is zero. This is an important proof of the efficiency of the algorithm. Mathematically, the best path is the path with highest some of scores, the path might have the highest sum of score while one of these scores is zero; one single zero score might not affect much the whole sum. If at some point, the score of the cell was zero because there is an encounter with an obstacle, it still can be in the path if the sum of all points on the path is still high. However, such situation is very rare as our algorithm and features are designed to minimize the chance of crossing obstacles; what prevents crossing obstacles is that normally there are other free cells with higher potential scores to choose instead of occupied cells. That’s why the probability to cross obstacles is almost zero. No encounter with obstacles will happen unless it is necessary and there is no other choice; in odd cases like the in the case of the divergence (stuck) problem that happens when the trajectory is trapped in a small area; the path might cross the wall to get out of the trap. To explain that, in some cases of divergence, the input location becomes so far from the current cell to be evaluated (big distance), when we calculate the feature (1/distance), and the probability (Equation (2)) it will be of very small value (near to zero). That means no big difference of probability between free cells and blocked cells; both would have very low scores because all are distant from the real place. In this case, it is possible that the path will cross an obstacle to get out from the trap, as again at the end the path is evaluated with the sum of probability along it not on a single obstacle cross. According to our observations, this case of crossing obstacles happens very rarely and does not happen in most of divergence problem cases, but few of them. The divergence problem itself happens rarely as we will see below. To study the effect of cell sizes and noise level, Table 2 was constructed with different parameter combinations. To measure the precision, the cumulative error for 50% and 90% of positions on the trajectory was calculated for different parameter values, in this case, cell size and noise level. For each parameter combination, the algorithm was run on 25 random input trajectories and the average cumulative error registered for 50% and 90% of positions.

**Figure 6:**
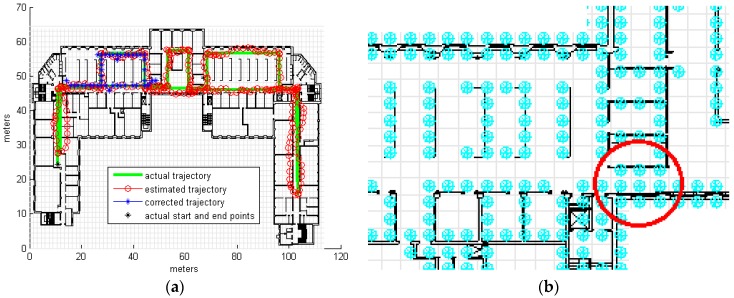
Large cells result in more cells being occupied by obstacles and blocked to movement, and corridors can be blocked. (**a**) shows a map matched trajectory for a cell size equal to 1.5 m; occupied cells are marked with blue circles (**b**) where we can see how the corridor that is marked by the red circle is blocked by obstacles.

**Figure 7:**
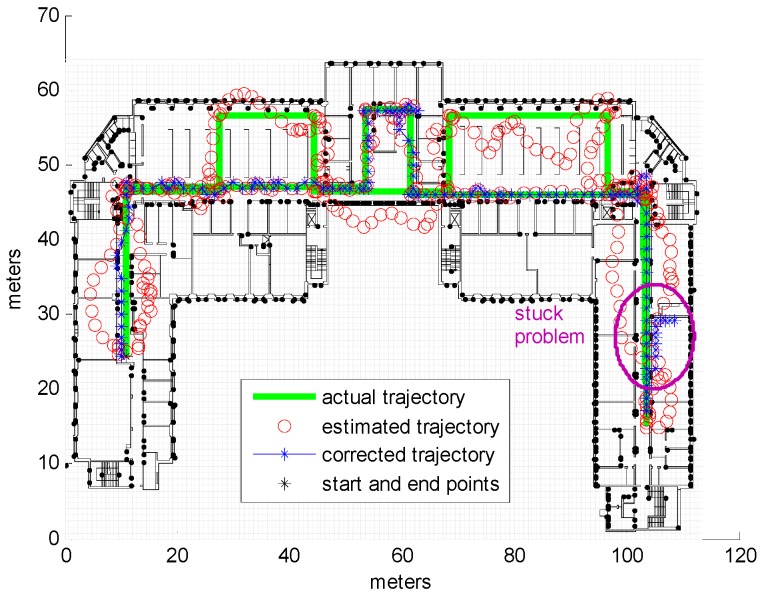
Stuck problem scenario, cell size = 0.8 m, noise level = 4.

**Figure 8:**
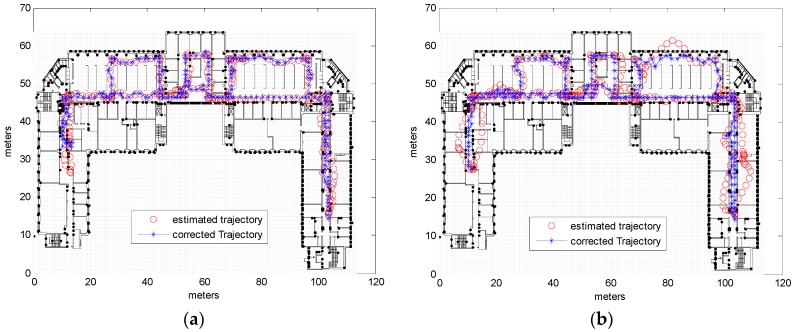

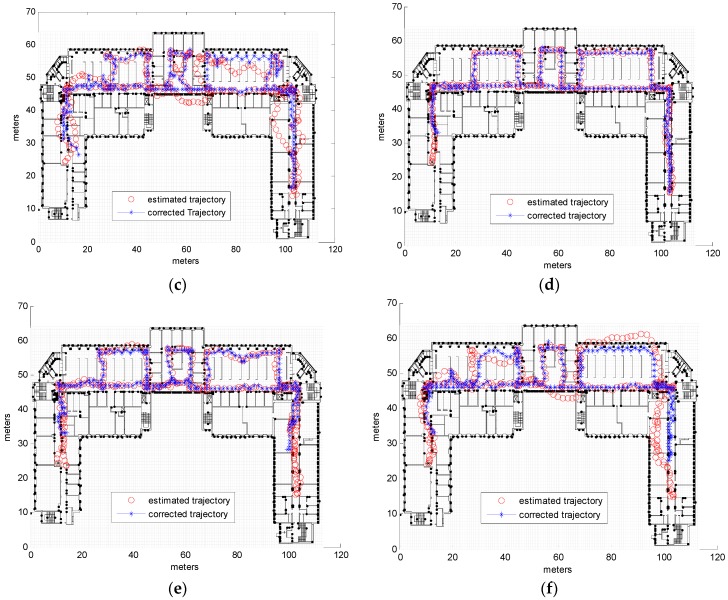
Ground truth, estimated and corrected trajectories obtained using different cell sizes and noise levels. (**a**) Cell size = 1 m, noise level = 2; (**b**) Cell size = 1 m, noise level = 3; (**c**) Cell size = 1 m, noise level = 4; (**d**) Cell size = 0.8 m, noise level = 1; (**e**) Cell size = 0.8 m, noise level = 2; (**f**) Cell size = 0.8 m, noise level = 3.

It is clear from Table 2 that a cell size of 1m provides good results, and even when adding high levels of noise to the input the results still acceptable. At a low noise level (equal to 1), for 50% of the trajectory the average cumulative error is 1.16 m and for 90% the average cumulative error is 3.078 m, based on a total track length of 352 m. Using a larger cell size of 1.5 m length results in more cells occupied by obstacles and thus blocked to movement; this explains the high level of error observed in the respective results, as illustrated by the example shown in Figure 6a. Figure 6b illustrates how the corridor is blocked because it contains cells occupied by side walls. Besides being high in error we also notice the results of this cell size are also totally random in relation with the noise level, higher error of low noise level is just due to the random input samples, using different samples would result in different numbers, that explains the available results of 70.7003 m using noise level 1 while smaller error 61.6751 m error is obtained using noise level 2, which might look strange but it is normal; noise level in this specific situation is irrelevant and does not have a significant impact on the results as the error that results from the blocked corridors is much higher than the error that results from the noise. We have run other samples and we got results which looks normal (smaller error for less noise level) but we kept the current result to illustrate the irrelevance of the noise level in this case. Although the use of a cell size of 0.8 m would be expected to lead to more precise results, the obtained results show that this is correct only for low noise input; at higher noise levels the error was high, likely due to what we call the stuck or divergence problem. This particular problem occurs when the corrected trajectory enters a room or small area and cannot leave; an example of such a situation is shown in Figure 7. Although with smaller cells it is more likely that certain cells will contain no obstacles, some of these empty cells could form entrances to small areas, allowing the trajectory to pass through them and become trapped. The stuck problem recorded in 0.8 m cell size simulations resulted in high error values in about 16% of samples, with the remainder achieving good results (average cumulative error of 4.2522 m for 90% of observations at a noise level of 2). It should be noted that the values shown in Table 2 indicate the average cumulative errors in all samples, including those experiencing the stuck problem, which explains the high value of some of these averages. Figure 8 displays examples of the results obtained for different cell sizes and different noise levels.

A moderate walking speed is thought to be around 1.67 steps/s (1.67 Hz) [20], with an average step length of 0.7 m for women and 0.78 m for men [21], resulting in a movement speed of around 1.3 m/s. Assuming a frequency of input of 1 estimation/s (1 Hz), i.e., a time step equal to 1 s, the subject would move 1.3 m every time step, 2.6 m in 2 steps, etc. As the proposed map model employs discrete values for movement (cell units), allowing only one cell transition, for example, in each time step (buffer size equal to 1 cell) would mean that the system would not be able to capture the movement of a pedestrian walking at moderate speed; it is therefore more suitable to have flexibility of movement by allowing 0, 1, or 2 cell moves in each time step by allowing transition to neighbor cells and to neighbors of neighbors (buffer size equal to 2), which is necessary in order to obtain a correct trajectory assuming a cell size of 1 m. If the input frequency was 2 Hz (2 estimations/s), this would mean an estimation every half-second time step. During this time period, a pedestrian moving at moderate speed moves around 0.65 m in each time step, a distance smaller than the cell size (1 m). In this case, if only one cell transition was allowed there should not be a problem, while allowing more possibilities (1 or 2 cells) should also work. In some systems there is no fixed frequency for estimations, with the location registered at each step whatever the speed. In the present case, if we assume a moderate walking speed the average frequency will be around 1.67 Hz, meaning that a buffer size of 1 cell would be suitable. However, in all cases a buffer size of 2 cells is still more flexible in capturing any speed variations and in dealing with other types of movement such as running.

The results shown in Table 2 are those for a buffer size of 2 cells and assuming that the estimations of the primary localization system are registered at a frequency of 1 Hz. To experimentally test the effect of buffer size and estimation frequency on the proposed algorithm, further simulations were carried out using a cell size equal to 1 and noise level equal to 1; the results of these simulations are shown in Table 3. It is clear from this table that the results were worse when using a small buffer size (1 cell) and 1Hz frequency, with 33.1054 m error for 90% of data compared to the 3.078 m recorded for a buffer size of 2 cells and the same 1 Hz frequency. This discrepancy is due to the inability of the trajectory to reach the correct destination using small steps, as the estimated trajectory was moving faster. Furthermore, the adoption of a 2 Hz measurement frequency achieved better results, even when using a small buffer. Nevertheless, a high buffer size always provides more flexibility.

### 3.2. Real Measurements

**Figure 9:**
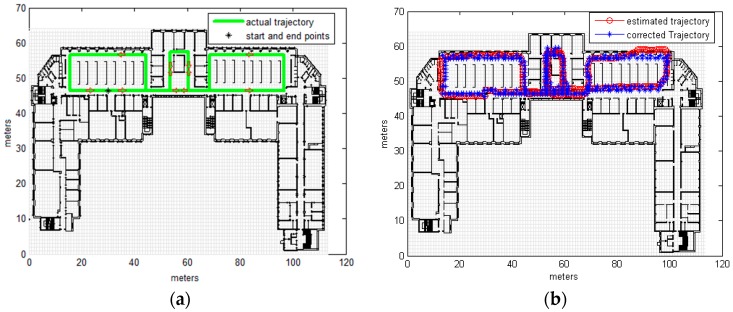

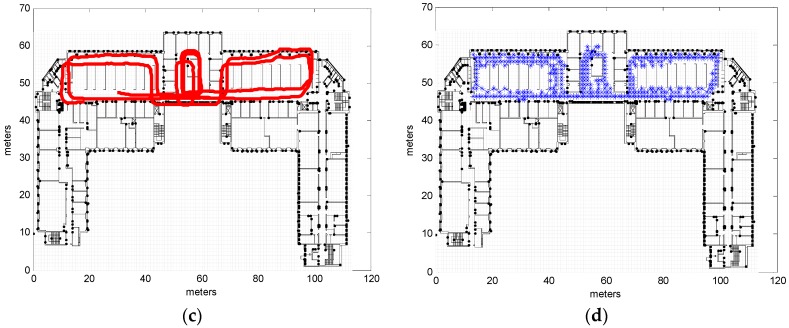
Ground Truth, estimated trajectory obtained from real measurements using iHDE and trajectory corrected using CRF. (**a**) Ground truth; (**b**) Estimated and corrected trajectories; (**c**) Estimated trajectory for two circuits; (**d**) Corrected trajectory for two circuits.

Testing was carried out on the 4th floor of the DeustoTech building at the University of Deusto (Bilbao, Spain). Primary results were estimated via a step-and-heading based Pedestrian Dead Reckoning (PDR) system using a wrist-worn inertial measurement unit composed of three accelerometers and three gyroscopes. Heading drift was reduced by applying the method known as improved Heuristic Drift Elimination (iHDE) [22], with the developed map matching algorithm then applied offline. Estimation frequency was not constant but depended on the step, as the employed PDR system calculated the location at each step. For the map model a cell size of 1m and a buffer size of 2 cells were used. The estimated results of the primary localization system and the results corrected by CRF map matching for one round walk of the DeustoTech area are shown in Figure 9b. Figure 9c displays the estimated trajectory for two rounds, with the corrected trajectory for this estimated trajectory shown in Figure 9d; the ground truth is shown in Figure 9a.

Analysis of Figure 9 reveals that although the primary estimated trajectory crosses a number of walls and obstacles, this was corrected successfully using the map matching algorithm and zero obstacles was crossed by the map matched trajectory, see Table 4. As a consequence of matching the trajectory with the map, the accuracy was also improved; even though the accuracy of the iHDE trajectory was already good, the map matching using CRF has further improved the accuracy and the cumulative error was decrease as shown in Table 4. Before applying the map matching, using only the primary localization system (iHDE), the average cumulative error was 1.2861 m and for 50% of the trajectory 2.6858 m for 90% of the trajectory. The cumulative error was reduced by applying the CRF map matching algorithm to be 1.0634 m for 50% of the trajectory, and 2.2316 m for 90% of the trajectory; which means improvement of 17.5% and 19.8%, respectively.

## 4. Conclusions

The presented research has demonstrated the successful use of CRF as a model in the offline map matching process. Our CRF algorithm is able to correct trajectories produced by localization systems when given the appropriate map information. Grid cells of fixed length were used for the map representation, with a cell size of 1 m producing acceptable results. The use of a larger cell size achieved less accurate results, mainly due to obstacles blocking entire cells, a feature unsuitable for sites with narrow areas such as corridors. On the other hand, although smaller cells should provide more accurate results, they also potentially increase the occurrence of the divergence problem that occurs when trajectories are stuck inside small semi-enclosed areas. Even though the experiments were carried out with pedestrians in mind, the same algorithm can be applied to any object moving within the normal pedestrian speed range; this is because the algorithm can be loosely coupled with any localization system and is independent of that system implementation, instead dealing with only its output coordinates.

Our future work will first involve finding methods with which to solve the stuck problem, possibly by adding more semantic information such as rooms and doors to the map model. In addition, using more context information and adding more algorithm features can help to further improve the results; people behavior related to the building structure can be another source of information that we intend to employ in order to improve the accuracy. Exploring ways in which to use the CRF model efficiently for online applications is another future task we will work on. 

## Figures and Tables

**Table 1:** Means and standard deviations for the different level of noise used in simulations.

Noise Level	Mean (m)	Standard Deviation (m)
1	1.3	1
2	1.6	1.1
3	2.0	1.3
4	2.5	1.8

**Table 2:** Simulated trajectory results using feature f_dis_. Path length = 352 m.

Cell Size (m)	Noise Level	Number of Crossed Obstacles	Cumulative Error (m)	
			50%	90%
0.8	1	0	0.9867	2.6699
	2	0	2.682	19.7820
	3	0	5.7945	32.464
	4	0	6.5158	32.3861
1	1	0	1.1574	3.078
	2	0	1.2463	3.8172
	3	0	1.4853	5.1879
	4	0	2.1409	11.1061
1.5	1	0	19.4403	70.7003
	2	0	16.6941	61.6751
	3	0	17.7461	68.5491
	4	0	20.0473	71.8129

**Table 3:** Simulated trajectory results for different step lengths and measurement frequencies using cell size = 1 m and noise level = 1. Path length = 352 m.

Estimation Frequency	Buffer Size	Cumulative Error (Meters)	
		50%	90%
1 Hz	1 cell	12.4931	33.1054
2 Hz	1 cell	1.1348	2.4989
1 Hz	2 cells	1.1574	3.078
2 Hz	2 cells	1.3639	3.0044

**Table 4:** Real measurement results using feature f_dis_ and cell size = 1 m. Path length = 347 m.

Algorithm	Number of Crossed Obstacles	Cumulative Error (m)	
		50%	90%
iHDE	15	1.2861	2.6858
iHDE + CRF	0	1.0634	2.2316

## Abbreviations

GNSS	Global Navigation Satellite System
CRF	Conditional Random Fields
HMM	Hidden Marcov Models
PDR	Pedestrian Dead-Reckoning
DXF	Drawing Interchange file format
CDF	Cumulative Distribution Function
iHDE	improved Heuristic Drift Elimination
